# Artificial Intelligence in Thyroid Field—A Comprehensive Review

**DOI:** 10.3390/cancers13194740

**Published:** 2021-09-22

**Authors:** Fabiano Bini, Andrada Pica, Laura Azzimonti, Alessandro Giusti, Lorenzo Ruinelli, Franco Marinozzi, Pierpaolo Trimboli

**Affiliations:** 1 Department of Mechanical and Aerospace Engineering, Sapienza-University of Rome, 00184 Rome, Italy; andrada.pica@uniroma1.it (A.P.); franco.marinozzi@uniroma1.it (F.M.); 2 Dalle Molle Institute for Artificial Intelligence (IDSIA), Università della Svizzera Italiana (USI), Scuola Universitaria Professionale della Svizzera Italiana (SUPSI), Polo Universitario Lugano-Campus Est, 6962 Lugano-Viganello, Switzerland; laura.azzimonti@idsia.ch (L.A.); alessandro.giusti@idsia.ch (A.G.); 3 Information and Communications Technology, Ente Ospedaliero Cantonale, 6500 Bellinzona, Switzerland; lorenzo.ruinelli@eoc.ch; 4 Clinical Trial Unit, Ente Ospedaliero Cantonale, 6500 Bellinzona, Switzerland; 5 Servizio di Endocrinologia e Diabetologia, Ospedale Regionale di Lugano e Mendrisio, Ente Ospedaliero Cantonale, 6900 Lugano, Switzerland; Pierpaolo.Trimboli@eoc.ch; 6 Faculty of Biomedical Sciences, Università della Svizzera Italiana (USI), 6900 Lugano, Switzerland

**Keywords:** thyroid neoplasm, medical imaging, artificial intelligence, machine learning, deep learning, radiomics, prediction, diagnosis

## Abstract

**Simple Summary:**

The incidence of thyroid pathologies has been increasing worldwide. Historically, the detection of thyroid neoplasms relies on medical imaging analysis, depending mainly on the experience of clinicians. The advent of artificial intelligence (AI) techniques led to a remarkable progress in image-recognition tasks. AI represents a powerful tool that may facilitate understanding of thyroid pathologies, but actually, the diagnostic accuracy is uncertain. This article aims to provide an overview of the basic aspects, limitations and open issues of the AI methods applied to thyroid images. Medical experts should be familiar with the workflow of AI techniques in order to avoid misleading outcomes.

**Abstract:**

Artificial intelligence (AI) uses mathematical algorithms to perform tasks that require human cognitive abilities. AI-based methodologies, e.g., machine learning and deep learning, as well as the recently developed research field of radiomics have noticeable potential to transform medical diagnostics. AI-based techniques applied to medical imaging allow to detect biological abnormalities, to diagnostic neoplasms or to predict the response to treatment. Nonetheless, the diagnostic accuracy of these methods is still a matter of debate. In this article, we first illustrate the key concepts and workflow characteristics of machine learning, deep learning and radiomics. We outline considerations regarding data input requirements, differences among these methodologies and their limitations. Subsequently, a concise overview is presented regarding the application of AI methods to the evaluation of thyroid images. We developed a critical discussion concerning limits and open challenges that should be addressed before the translation of AI techniques to the broad clinical use. Clarification of the pitfalls of AI-based techniques results crucial in order to ensure the optimal application for each patient.

## 1. Introduction

The role of medical imaging in the clinical workflow has noticeably increased from a mainly diagnostic tool up to a central contribution in early detection, diagnosis, treatment planning and monitoring of diseases [1,2,3,4]. Medical imaging provides information concerning the characteristics of human tissues in a non-invasive, repeatable manner and became a routine practice in clinical care [2]. In recent decades, the innovations in this field concerned both devices, i.e., hardware, and analysis tools used in medical imaging. In the clinical practice, the main use of medical images corresponds with qualitative assessment of the anatomical area. Images, in addition, are characterized also by a high quantity of numerical information and recently, a quantitative evaluation has been developed in order to identify possible correlations between the numerical data contained in the digital images and the pathophysiology of the tissue [3]. The quantitative analysis has the aim to achieve information from standard-of-care images, e.g., ultrasound imaging (US), computer tomography (CT), magnetic resonance imaging (MRI) and positron emission tomography (PET), which are not easily quantifiable by means of naked-eye observations for clinical outcomes [5,6].

Analysis of image features in the context of medical imaging is an emerging field of study but extensive literature already exists [7,8,9]. In the majority of earlier works, the image features are analyzed with the aim of detection and diagnosis of abnormal regions within human tissues [10,11,12]. These applications are often referred as computer-aided detection (CADe) and computer-aided diagnosis (CADx) systems [3]. The output of the CAD analysis is used by the expert clinicians as a second opinion in detecting lesions or making diagnosis and aims at improving the accuracy of the diagnosis and reducing the time for image interpretation [6].

Recently, a further detailed extension associated with quantitative analysis of medical imagines has led to the emergence of radiomics as a new field of medical research [1,2]. Radiomics aims at extracting numerous quantitative descriptors with the purpose of achieving more useful information of tissue lesion and response of treatment in order to be used for personalized medicine [1,2,13]. It is worth noticing that standardization of the procedure is still under development, as thoroughly discussed in [14].

The above-mentioned approaches apply methodologies from the artificial intelligence (AI) field to achieve a partial or full automation of various steps of the process concerning the analysis of medical images [6]. Thorough understanding of their working principle is necessary in order to develop efficient predictive models and personalization treatment. This review article aims to highlight strengths and limitations of the different AI-based techniques applied for the evaluation of the pathophysiological state of the thyroid.

## 2. Artificial Intelligence in Medical Imaging

Artificial intelligence (AI) is a term coined by McCarthy and colleagues [15,16] in 1950s referring to a branch of computer sciences in which mathematical algorithms attempt to perform tasks that normally require human cognitive abilities [8]. Applications of AI have witnessed unprecedented growth in recent decades due to the enhancement of computational power and availability of large dataset. In the medical field, AI can use complex algorithms to develop models with the scope of improving diagnostic accuracy, prognosis, and medical image interpretation [17]. We discuss in the following two different machine learning (ML) methodologies adopted to perform medical imaging analysis.

### 2.1. Machine Learning

Machine learning (ML), a term first coined by Arthur Samuel [18], is a field of AI in which the computer is trained to perform tasks by learning from example data and make predictions based on its exposition to previous samples [4]. In medical imaging analysis, ML algorithms are crucial components of both CAD systems and radiomics studies.

ML algorithms are generally divided into supervised and unsupervised learning methods. Supervised learning requires a labelled dataset, i.e., a set of input data with their corresponding output (labels) that is used to identify a function linking inputs to outputs [19]. Unsupervised learning operates on an input dataset without the need of labels. This ML algorithm searches for patterns that can separate input data into subsets with similar characteristics [7]. In this review article, we focus on supervised learning since it is the most common approach applied to medical images analysis [20].

In medical applications, input data include medical images or clinical data, while the output label can be the differentiation of malignant from benign nodules, the classification of images into diagnostic categories or the response treatment, e.g., recurrence, survival. The output of the predictive model leads to a subsequent distinction of ML problems: classification and regression. In classification tasks, the model performs a decision among a small and discrete set of choices, i.e., binary classification, e.g., identifying a tumor as malignant or benign. Regression models refers to the estimation of continuous output variables, e.g., assessment of disease severity [20].

**Figure 1:**
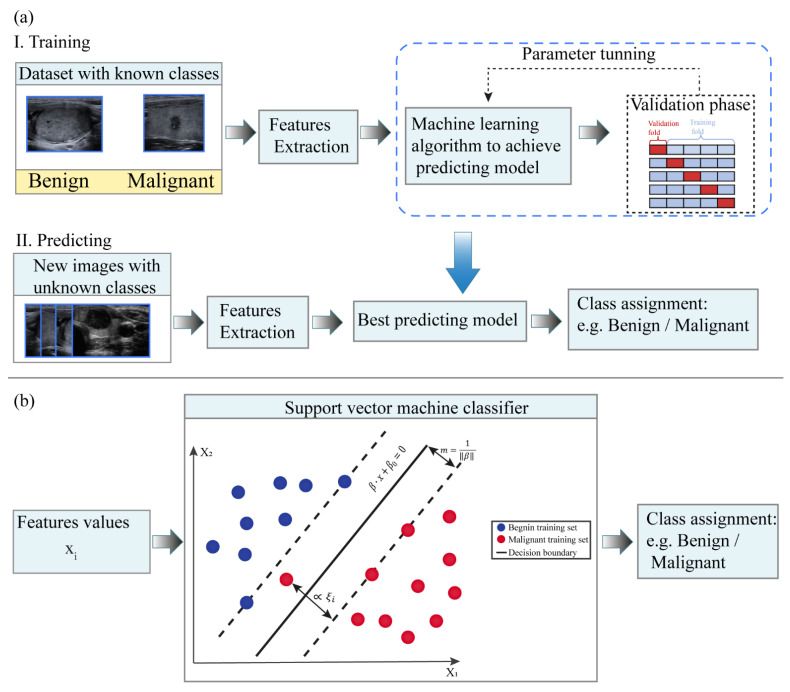
(**a**) Schematic flowchart of the machine learning model implementation and application for medical images classification purposes. (**b**) Example of the support vector machine (SVM) classification with a hyperplane that maximizes the separating margin m between the two classes.

Historically, ML algorithms were applied in CAD systems for classifications purposes [20]. Subsequently, this method was used as a step of radiomics analysis. In this section we describe the workflow of the ML algorithm with classification task frequently encountered in the CAD framework [10,21,22] (Figure 1). A supervised ML model is composed of two phases, i.e., training and application phase (Figure 1a). In the training phase, a set of input images with their corresponding class labels are used to train the predicting model. From the input image, a region of interest (ROI) is delineated manually or semi-automatically by expert clinicians. Subsequently, a set of image features, e.g., morphological and grey level-based features, are extracted. Differently from other methods that will be discussed subsequently in this work, in ML algorithms of CAD systems, the extraction and selection of image features are performed manually by the expert. It represents a crucial step in order to identify the significant variables that can be correlated with the medical endpoint. In CAD applications, the features used in the analysis are those closely associated with what clinicians use in their diagnosis of the lesions [4]. Subsequently, the features are entered as input to the ML algorithm to train the model.

Examples of typical feature-based supervised learning algorithms are logistic regressions, support vector machine, random forests and neural networks [23]. As an example of these feature-based ML algorithms, we focus here on the support vector machine (SVM) method, which is commonly used in biomedical binary classification problems [17,24]. Overall, SVM (Figure 1b) is a binary classifier that aims to identify the decision boundary, or hyperplane, that maximizes the separating margin between two classes [4,25].

For instance, let consider N training samples ${\left\{\left(x_{i},y_{i}\right)\right\}}_{n=1}^{N}$ of input features *x* and their corresponding class or label *y_i_* ∈ {−1;+1} where *y_i_* = −1 indicates the class with malignant samples and *y_i_* = 1 indicates the class with benign samples. In the simplest case, there exists a function *f*(*x*):

(1) $$f\left(x\right)=\beta \cdot x+\beta_{0},$$

with *β* and *β*_0_—decision boundary parameters such that *f*(*x*) ≥ 0 for *y_i_* = +1 and *f*(*x*) < 0 for *y_i_* = −1.

This means that the training samples from the two classes are separated by the hyperplane *f*(*x*) = *β*∙*x* + *β*_0_ = 0. The margin m, i.e., the distance between a class and the decision boundary, is set to be inversely proportional to the decision boundary parameter, i.e., $m=\frac{1}{\left\|\beta \right\|}$.

In order to identify the hyperplane that maximizes the separating margin between the two classes, SVM solves the following optimization problem that aims to minimize the cost function *J*(*β*, *ξ*) with respect to *β*, *ξ* [24,26]:

(2) $$\underset{\beta ,\;\xi }{\min}\;J\left(\beta ,\xi \right)=\underset{\beta ,\;\xi }{\min}\frac{1}{2}{\left\|\beta \right\|}^{2}+C\overset{N}{\underset{i=1}{\sum }}\xi_{i}$$

subject to the constraints *y_i_* (*β*∙*x* + *β*_0_) ≥ 1 − *ξ_i_*, *ξ_i_* ≥ 0, *i* = 1, …, *N*. In Equation (2), *C* > 0 is a penalty parameter to control the tolerance error *ξ_i_* allowed for each sample being on the wrong side of the margin.

From Equation (2), it can be noticed that the minimization of the parameter *β* increases the separation between the two classes and improves generalizability of the classifier, while minimization of second term of Equation (1) improves fitting accuracy [4].

Subsequently, in the testing phase, the trained classifier is used to characterize new input data with unknown label (test set).

It is worth pointing out that the decision function of the classifier is fully specified by the training set, while the test set is only used to evaluate the performance of the model. On one hand, to obtain a model that well-performs when applied to new data, the training dataset should be sufficiently large. On the other hand, to obtain robust and reliable evaluation of the performance of the model, the test set should be sufficiently large. Frequently, since this condition is difficult to achieve in the medical field by simply splitting the available data in training and test set, a k-fold cross-validation framework [7] is usually adopted. K-fold cross validation consists of partitioning the dataset into k subsets of equal size. The model is trained on (k − 1) datasets while one subset is retained for model test. The process is repeated k times with each subset used once as test dataset [20]. The overall performance of the model is then assessed for example as the average performance over the k repetitions.

Feature-based ML algorithms are suitable for medical image analysis since predictive models can be developed from small datasets [7]. Moreover, these methods are usually interpretable and can provide insights on the reasons why a certain class is predicted. Nonetheless, some initial steps of the process, as the definition of the features to be extracted from images and the selection of the medical region of interest has to be performed by experts. In addition, it should be taken into account that all supervised ML methods could be affected by overfitting, i.e., the predicting model learns exactly the training set but fails to fit new data from the test set [20]. However, it is possible to mitigate this issue by adopting a cross-validation set-up and by reducing the number of features used by the model by means of feature selection methods.

### 2.2. Deep Learning

Deep learning (DL), a term coined in 1986 by Rina Dechter [27], is a new class of ML methods developed through the advancement of artificial neural networks which were considered as artificial representations of the human neural architecture [23]. DL relies on networks of computational units, i.e., neural units arranged in layers that gradually extract higher level features from input data, e.g., image. These structures learn discriminative features from data automatically, allowing to approximate complex nonlinear relationship with outstanding performance [27,28]. Differently from traditional feature-based ML approaches, DL is able to achieve diagnosis automation, avoiding human intervention [29]. In medical applications, DL algorithms are implemented for detection and characterization of tissue lesions as well as for the analysis of disease progression [27,28].

While several DL architectures have been developed, this article focuses on convolutional neural networks (CNNs), introduced by LeCun [30]. CNNs are typically applied for image recognition and computer vision applications because they preserve spatial relationships in 2D data, and therefore outperform other architectures on image pattern recognition. More specifically, the input of a CNN is arranged in a grid structure and processed through convolution and pooling layers that preserve these relationships. The final layers are typically fully connected and can be conceived as a multi-layer perceptron classifier on the features automatically extracted by the convolutional part. The network is trained to identify patterns in a set of labelled training data and the outputs are compared with the actual labels. During training the network parameters are tuned until the patterns identified by the network represent good predictions for training data. The network is then used to make predictions on new data in the test set [31].

**Figure 2:**
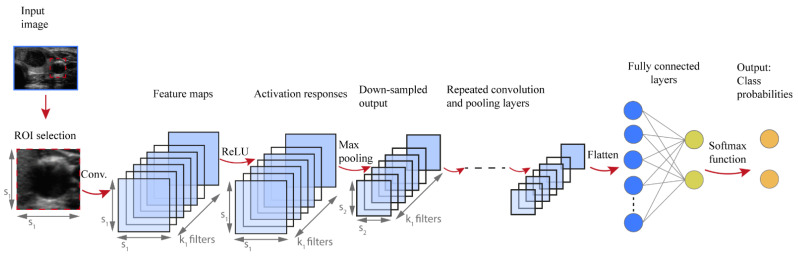
Schematic flowchart of the deep learning model implementation and application for medical images classification purposes.

**Figure 3:**
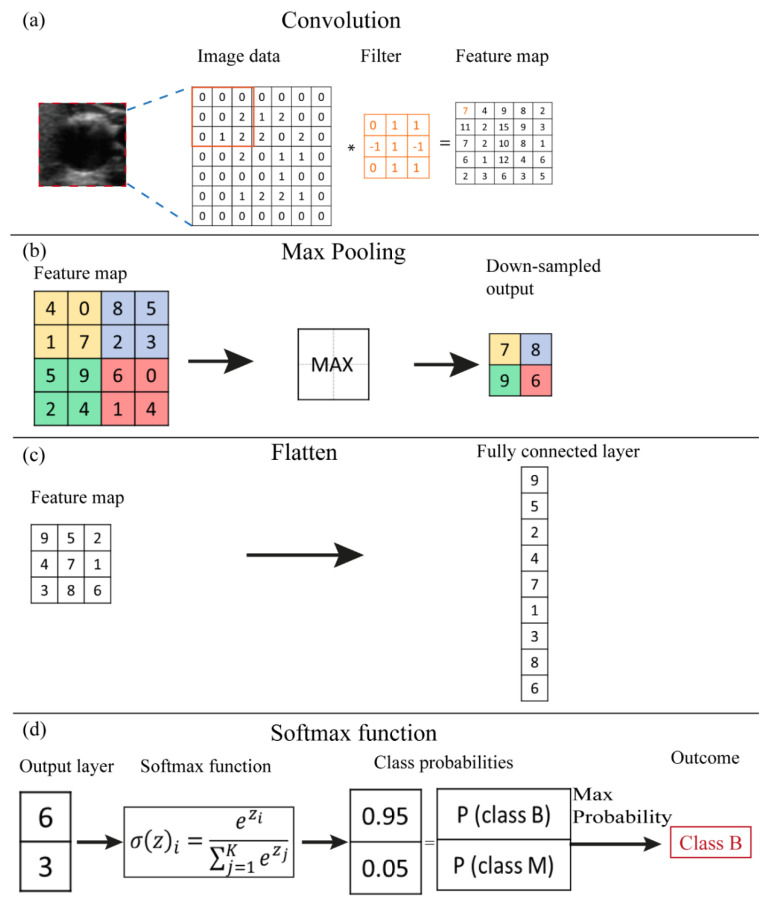
(**a**) Numerical example of the functions that compose the CNN architecture: (**a**) convolution, (**b**) max pooling, (**c**) flattening, (**d**) softmax function.

Figure 2 shows a typical architecture of CNN developed to perform classification tasks. The input of the CNN algorithm is represented by numerical data of the selected ROI from the medical image. Firstly, a convolutional step is considered which contains a set of filters, e.g., k_1_ in Figure 2. Thus, a convolution is performed between each filter and the input of the layer, e.g., image data. A convolution is a space-invariant linear operation on 2D grids and is equivalent to applying a filter to an image. The filter slides over the input image, its values are multiplied with the image pixel values and then summed to determine the value in the corresponding position of the output feature map. An example of a convolution operation is reported in Figure 3a. The number and size of filters are CNN hyperparameters and are typically not optimized during training. More and larger filters lead to more powerful network with more parameters to optimize, which increases the risk of overfitting [32]. The convolutional process in every convolutional layer is expressed mathematically as follows:

(3) $$X_{k}^{\ell }=\sigma \left(W_{k}^{\ell -1}*X^{\ell -1}+b_{k}^{\ell }\right)$$

where $X_{k}^{\ell }$ is the new feature map, σ(∙) is an element-wise nonlinear activation function, *W* is the filter values, $b_{k}^{\ell }$ is a bias parameter and the symbol ∗ indicates a convolutional operator.

Subsequently, an activation function is applied element-by-element to the calculated output of the convolution prior to using the map as an input to the next layer of the network. Rectified linear unit (ReLU) is one of the most used activation functions, and has been empirically found to accelerate the convergence of the learning procedure [28]. It is linear for positive inputs, mapping them unchanged to the next layer, while it blocks negative values. Mathematically, ReLU is expressed as follows [28]:

(4) f (x)=max(0, x)

where *x* is an activation value achieved from the previous layer.

Some CNN architectures also consider pooling operations, whose effect is to downsample the feature maps. This operation considers small regions of the input map and outputs a single number for each region, e.g., the maximum value as illustrated in Figure 3b. It reduces the dimensions of the feature map and decreases the number of pixels to be processed in the next layers of the network [33]. Conceptually, as we progress deeper in the network, neuron activation values represent progressively higher-level and larger-scale visual patterns in the input, and therefore require lower spatial resolution.

The final part of the CNN architecture is characterized by a fully connected layer, i.e., each neural unit of the actual layer is connected to every neural unit in the successive layer (Figure 2). Firstly, the feature map is flattened into a column vector (Figure 3c) and then connected to one or more fully connected layers. The output nodes of the last fully connected layer can be regarded as a vector of unnormalized probabilities [28].

The softmax function is a function applied to the last fully connected layer of the CNN in order to transform the k real values of the vector into values in the range (0;1) so that can be assumed as probabilities (Figure 3d). The relation is as follows [28,33]:

(5) $$\sigma {\left(z\right)}_{i}=\frac{e^{z_{i}}}{\sum_{j=1}^{K}e^{z_{j}}}$$

where the *z_i_* values are the elements of the fully connected layer and the denominator represents the normalization term.

The output layer of the CNN considered is constituted by neural units which indicate the probabilities for each class.

The analysis of the available literature shows an increasing interest on applying DL architecture for medical image analysis. It is worth mentioning that for systems in which the set of visual features is well defined, simpler feature-based ML techniques, such as SVM algorithms, are easier, more interpretable and more effective [28].

The main limitation to the use of DL consists of the large datasets required to train the model [34]. Compared with publicly available datasets in other areas, the current availability of medical US datasets is still limited [34]. To face the data requirements, several studies [33,35] considered pre-trained CNN architectures developed with trainings on ImageNet, a large labelled collection of low-resolution color photographs. To date, DL architectures pre-trained on high resolution medical images are not available. Therefore, a large dataset of medical images is a mandatory step to enhance CNNs performance [34].

## 3. Radiomics

Radiomics is an emerging field that uses automated high-throughput extraction algorithms to achieve large amounts (200+) of quantitative features from medical images [1,2]. Radiomics is also indicated as quantitative imaging [36] which can be applied to any image generated in the clinical setting. It can be performed on subregions of a tumor, metastatic lesions and in normal tissues. The term feature represents a descriptor of an image, of tumor or healthy tissue, such as parameters derived from image grayscale intensity or shape [37].

Radiomics has its roots on computer-aided diagnosis systems [38], although methodological workflow and applications are distinct [2]. It concerns the extraction of quantitative features from medical images that subsequently are related to biological endpoints and clinical outcomes [39]. Radiomics makes use of digital data stored in those images to develop diagnostic, predictive or prognostic models to support clinical decisions and optimize personalized treatment planning. The main difference with CAD systems consists of the relationship that radiomics has to identify between the current characteristics of the tissue lesion and its temporal evolution in the perspective of a personalization of the therapy [38].

**Figure 4:**
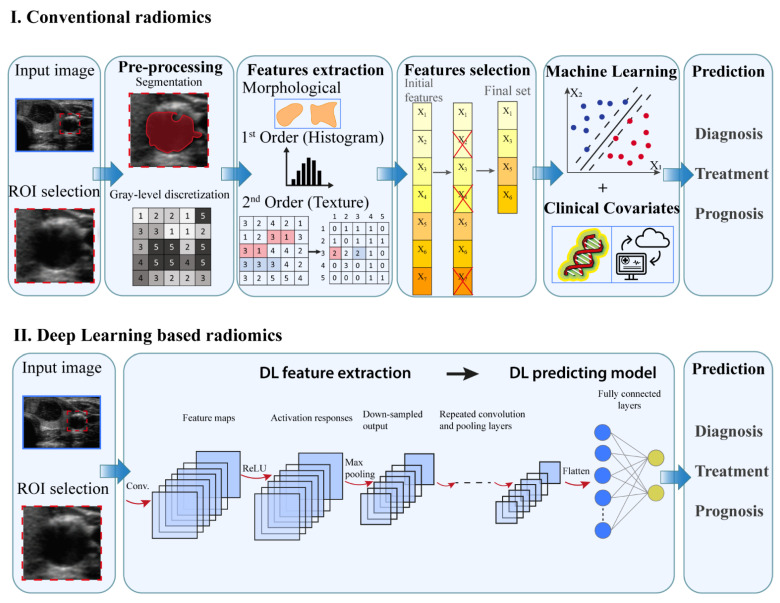
Schematic flowchart of radiomics approach. X_i_ represent the feature extracted from the image data.

Radiomics involves several processes, each with its own critical aspects that need to be taken into account. Two workflows can be implemented to perform radiomic studies in function of the AI technique adopted (Figure 4): (i) conventional or ML-based radiomics where the features to be extracted are predefined and (ii) DL-based radiomics where the features are not predefined but automatically extracted from the underlying data [6,7].

The main aspects of the conventional radiomics workflow concerns: image acquisition, data selection, feature extraction and selection and the development of predictive model [1,36]. From medical image such as US, CT, MR and/or PET images, the region of interest (ROI) is selected and subsequently the lesion is manually segmented, i.e., delineated with computer-assisted contouring, by an experienced clinician [7]. Subsequently, image data undergoes preprocessing operations, e.g., gray-level discretization, which enable a higher reproducibility of results [6]. The extraction of quantitative imaging features involves descriptors of spatial relationships between the various intensity level, heterogeneity patterns, shape and relations of the tissue lesion with surrounding tissues. A feature selection procedure is then performed to identify the most relevant predictive features [7,24]. The collection of features which hold prognostic or predictive value represent a feature signature, frequently indicated also as quantitative imaging biomarkers. The selected features are then analyzed to develop classified models to predict outcomes either alone or in combination with additional information, such as demographic, clinical, comorbidity or genomic data [1,3].

Segmentation represents a crucial subprocess of radiomics since many extracted features may depend on the segmented region. In several radiomics studies the ROI is manually delineated by experts [21,40,41,42]. A number of algorithms has been developed for semi-automatic segmentation [22]. Region growing-based algorithm and grey-scale threshold-based methods are frequent techniques applied for ROI definition. However, manual delineation by an expert is considered the gold standard though is subjected to inter-observer variability and is a time-consuming task [37]. To avoid possible bias, evaluation by multiple clinicians or a combination of multiple algorithms could be considered [43].

Typically, radiomics features are divided into [2,6,44]:

1. Morphological, that are based on the geometric properties of the ROI, e.g.: volume, maximum surface area, maximum diameter.
2. First-order statistics or histogram based, which describe, through histograms, the distribution of grayscale intensity without concern for spatial relationships within the ROI. For instance, calculated features are grey level mean, maximum, minimum and percentiles.
3. Second-order statistics or textural features, that represent statistical relationship between the intensity levels of neighboring pixels within the ROI that allow to quantify image heterogeneity, e.g., absolute gradient, grey level co-occurrence matrix (GLCM) grey level run-length matrix (GLRLM), grey level size zone matrix (GLSZM) and grey level distance zone matrix (GLDZM). For instance, GLCM indicates the number of times the same combination of intensity occurs in two pixels separated by a specific distance δ in a known direction.
4. Higher-order statistics features, which are computed after the application of mathematical transformation and filters that lead to highlighting repeated patterns, histogram-oriented patterns or local binary patterns, e.g., wavelet or Fourier transforms.

Accurate definitions of radiomics features are provided in the image biomarker standardization initiative (IBSI) [14].

The radiomic features are subjected to a subsequent feature selection to prevent overfitting, improve learning accuracy and reduce computation time. The selection process should eliminate unreliable, not informative or redundant features. The selection methods can be divided into three classes: (i) filter methods which asses the usefulness of a given feature with various statistical tests for their correlation with the outcome variable [2,7]; (ii) wrapper method which uses an external classifier algorithm to score different subsets of features based on their classification performance; (iii) embedded method where the selection is intrinsic to the model training, i.e., features are selected to optimize the performance of the implemented learning algorithm. Filter methods are simple and computationally efficient, but consider features as independent and any interaction between them is ignored [24]. Wrapper methods reduce the risk of overfitting but are computationally intensive [7,24]. Embedded methods are computationally more efficient since the selection procedure is part of the training process [7,24]. A frequent embedded algorithm with good performance used in radiomics studies is the least absolute shrinkage and selection operator (LASSO) [7,24].

Subsequently, the selected features are used to implement a mathematical model in order to predict the established medical endpoints. Regarding the choice of modelling methodology, the identification of a suitable method depends on several factors as sample size or study endpoint [36]. It is advantageous to include in the model information beyond radiomics, e.g., clinical data and/or other “-omic” information, e.g., genomic data [45]. The integration of data from multiple sources, e.g., medical imaging, disease risk factors, therapy procedures and follow up data, in the mathematical model will facilitate the development of a personalized treatment.

As previously mentioned, the target of the radiomics studies can be either a present characteristic, e.g., tumor phenotype, or a future prediction, e.g., treatment response. Usually, radiomics studies make use of the feature-based ML algorithms that are also considered in CAD systems. By means of feature-based ML methods, the relationship between input data, e.g., selected radiomics features and target outcome, is determined by means of training examples. SVM is one of the most successfully applied algorithms.

DL-based radiomics allows to automatically extract imaging features and achieve the predicted outcome. In fact, the different components of the DL architecture perform all the processing steps described in the ML-based model, including feature extraction, selection and predicting model implementation. CNNs is the most common architecture used in radiomics studies and its characteristics have been previously described in Section 2.2.

Validation is a crucial component of the workflow of both conventional and DL-based radiomics. Ideally, the trained model should be tested in cross-validation or on an external, independent dataset before being applied on the new dataset [38].

## 4. AI and Radiomics in Thyroid Diseases

Ultrasound imaging is the recommended method for early detection and diagnosis of thyroid lesions due to its economy, effectivity and absence of radiation [46,47,48,49]. It is widely accepted as the first imaging modality for thyroid disease, for instance by American and European associations of endocrinology [50]. AI applications in the medical field are of increasing interest since they represent a possible approach to reduce the number of invasive clinical procedures [36].

Mainly, AI algorithms have been implemented for the classification of thyroid nodules, i.e., differentiating among benign or malignant state [9,10,21,22,33,41,51,52,53,54,55,56]. The outcomes of these studies are compared with the diagnosis of radiologists with different levels of experience. Research comparing the diagnostic ability between feature-based ML and DL algorithms is limited in the literature, but interesting outcomes are provided in [22]. Overall, an improvement emerged in terms of both specificity and accuracy in DL studies [57,58] with respect to feature-based ML classical applications [22], mostly determined by the capacity of DL of capturing complex patterns. In some studies [57,58,59], DL algorithms show accuracy values in line with those of radiologists. In addition, Jin et al. [20] also pointed out that the use of AI algorithms was useful to junior radiologists allowing a noticeable improvement of their diagnostic performance, reaching values of accuracy similar to those of intermediate-level radiologists. Studies of interest concerning the application of feature-based ML methods and DL algorithms are described in Table 1 and Table 2, respectively. Tables were organized according to the publication time, in a decreasing order.

Radiomics is considered a promising method to be encompassed in the pipeline of precision medicine on the basis of specific characteristics of the patient [2]. Whilst the first AI approach to the medical imaging, i.e., CAD system, is focused on the differentiation among benign and malignant thyroid lesions, radiomics extends the analysis to prognosis and response to treatment evaluation [1]. In fact, [42,60,61] implemented radiomics models that analyze the risk stratification and predict the aggressiveness of the thyroid carcinoma with high values of accuracy, i.e., roughly 85 percent. Radiomics analysis has the potential to determine tumor phenotypes or the presence of gene mutations [62,63]. Furthermore, several studies have investigated by means of radiomic features the occurrence of metastases [64] or disease-free survival [65]. It also emerged that radiomics studies aimed at performing classification tasks regarding the nature of thyroid nodules are characterized by minor accuracy with respect to classical ML approach [66]. It is worth pointing out that although radiomics has been applied for several anatomical areas, research concerning thyroid lesions is relatively limited. Studies of interest concerning radiomics applications for thyroid lesions are described in Table 3, organized according to the publication time, in a decreasing order.

## 5. Discussion

Medical images provide a comprehensive view of the tumor and its environment, and they can be used to improve the diagnostic accuracy of early lesions, to classify benign from malignant tissues and to define risk and improve therapy [43,68]. Imaging is a non-invasive method and with no risk of the infections or the complications that accompany biopsies [2]. In recent decades, images have been converted into quantitative data and subsequently analyzed with AI tools.

Intratumoral heterogeneity and modifications over time are common features of neoplasms [43]. Samples of tumor acquired through biopsy may fail to represent the variations within the tumor. In addition, AI methods, analyzing the overall image of the lesion, have the potential to capture tumor heterogeneity and could represent an intermediate step between imaging and biopsy [28,36]. Nonetheless, it is worth pointing out that AI systems learn on a case-by-case basis. AI algorithms are implemented considering gold standards of pathological diagnosis that are hard to identify in every patient, due to inter-variability among subjects. Moreover, as it emerged from the overview of the AI methods, the predicting model is developed on the basis of a finite training dataset. Thus, since human tissues are characterized by high heterogeneity and variability inter- and intra- subjects, no finite training set can fully represent the variety of cases that might occur in the clinical practice. Extensive research is still required to improve the generalizability and accuracy of AI-based models. From this perspective, the standalone use of AI applications for diagnosis should be still avoided in the clinical practice. In fact, to this date, several studies [7,20,28,43] recommend that the lesion evaluation should be achieved from a combination between the clinician evaluation and ML or DL outcome. Moreover, it is worth noticing that most AI-based studies focused on thyroid pathologies are performed using retrospectively collected data [9,11,33,40,42,51,55,60,61,62,63,65,66,67]. Conversely, studies that prospectively evaluate AI predictive models concerning thyroid disease diagnosis are limited in the literature [22,41]. In retrospective studies, cohorts are selected among patients with definitive diagnosis achieved mainly through histopathological examination. As highlighted by Wu et al. [69], evaluations should include more prospective studies on medical AI models to reduce risk of overfitting and enhance accuracy of the clinical outcomes.

AI methods are based on the analysis of image features in order to develop predictive models. Differentiating benign and malignant thyroid nodule is mainly achieved from ML-based studies. The most used US features adopted by ML algorithms for thyroid investigations were size, shape, margin, composition echogenicity, as defined by the thyroid imaging reporting and data system (TI-RADS) classification [10,21,22,51]. According to an analysis of the available literature, the TI-RADS approach allows a good discrimination among benign and malignant thyroid nodules. However, the inclusion of additional features, e.g., calcifications, internal content, can represent a factor that improves accuracy [70].

Radiomics studies were applied also to other thyroid pathologies, e.g., extrathyroidal extension (ETE) in patients with papillary thyroid carcinoma (PTC) [42,61], thyroid cartilage invasion from laryngeal and hypopharyngeal squamous cell carcinoma [64]. In these studies, the extracted features derive from morphological, first order statistics, textural and higher order statistics groups. Wang and colleagues [42] highlighted that improvement of ETE diagnosis is achieved when features related to PTC heterogeneity are taken into account. Similarly, in [64] Guo et al. studied thyroid cartilage invasion from laryngeal and hypopharyngeal squamous cell carcinoma and showed that tumor invasiveness can be investigated considering features related to tumor heterogeneity. Furthermore, Kwon et al. [62] highlight that BRAF mutation may be investigated with histogram-based and textural features that reflect echogenicity and heterogeneity of the region of interest, respectively.

Several studies also performed comparison between the performance of AI-based models and that of expert clinicians. The available data in literature mostly report that the performance of DL algorithms is similar to that of healthcare professionals. As discussed by [20,67], AI applications may improve the accuracy of thyroid diagnosis diseases, especially for junior radiologists. In fact, interpretation of medical images highly depends on the experience level of clinicians. For instance, for junior radiologists the sensitivity is reported in a range between 40 percent and 100 percent while the specificity spans between 50 percent and 100 percent. It was observed that the use of AI algorithms to achieve a second opinion on the characterization of thyroid lesions can improve the accuracy of junior radiologists from roughly 82 percent to 87 percent [67]. Moreover, Peng and co-workers [67] highlighted that taking into account the outcomes of AI as a second opinion has reduced fine needle aspiration procedures by 27 percent and the number of missed malignancies of roughly 2 percent.

Furthermore, the experience level of the clinicians has an important impact also on the performance of the AI-based methods. The input data of the AI algorithms is the ROI selected by the expert. It is commonly accepted that image acquisition and segmentation are critical subprocesses due to inter-operator variability. Recent studies [8,28] suggest that semi- or fully automated methods could improve algorithm performance, but currently the manual segmentation performed by experts continues to be the main method adopted. For instance, most of the ML-based studies applied to the thyroid are performed considering a manual segmentation of the ROI [21,41]. In addition, the ML-based investigations reported in [10,22] have introduced a semi-automatic method that is characterized by an initial automatic selection of a box region and subsequently by a manual contouring performed by expert clinicians. Conversely, the studies that applied DL algorithms to thyroid imaging considered a manual selected box around the region under investigation [9,11,52,54]. Furthermore, it is worth pointing out that radiomics studies are based on a manual contouring along the borders of the thyroid tumor [60,61,62] or slightly within the borders of the tumor to avoid artifacts [64].

To date, most studies highlight that the main limitation of AI algorithms is the reduced dataset used for predictive model development and validation. Ideally, independent training and validation datasets, composed of data images achieved with different US equipment and from multiple centers, i.e., multicenter training cohorts, allow to optimally develop the predicting model, avoiding overfitting and enhancing generalizability and model performance [67].

For instance, in radiomics studies, Gilies and coworkers [43] provide an empirical rule concerning the size of the dataset in order to avoid overfitting. It is suggested that almost 10–15 patients are needed for each examined radiomic feature. Thus, also features selection represents a crucial step during the evaluation.

AI methods represent a powerful approach that in future may assist clinicians in diagnostic decisions [22,71], while combined with other “-omic” data as occur in radiomics analysis may improve the risk factor analysis for personalized estimation of disease-free survival. As mentioned, AI methods could be also applied to contribute to treatment planning. For instance, radiomics combined with other clinical parameters may help to predict which patients are likely to have a satisfactory response to emerging therapies as high-intensity focused ultrasound (HIFU), that allows the thermal tissue treatment and the consequent reduction in thyroid nodule volume by directing energy inside the target zone with non-invasive instruments [72,73,74].

Several efforts are performed to increase the availability of open access database of labeled medical images that will help to train the predictive models developed with AI techniques. However, pitfalls and limitations associated with the AI approach should be considered, especially related to the difficulty to achieve a generalizable model in order to ensure optimal application for each patient.

With regard to the application of the AI in the daily practice of the clinical medicine, beyond the hype around these technologies, the financial investment is pouring and brand-new products started flowing into the market. As of early 2020, there were 64 FDA-approved AI-ML medical device and algorithms, many of which are already integrated into clinical care. Remarkably, 21 were related to Radiology [75]. Nonetheless, recent literature reviews report that the impact is still minimal as the majority of the AI-ML studies are retrospective in nature, deviate from existing reporting standards and often outline proof-of-concept approach [76].

From the pure clinical standpoint, all these findings should be interpreted according to the routine clinical practice. In fact, US is recognized as the most relevant imaging procedure for the assessment of thyroid nodule and almost all thyroid patients are managed according to US features of their thyroid gland. This worldwide diffused approach is based on the high sensitivity and specificity of US in discriminating malignant from benign thyroid lesions. Further improvement of US performance by AI remains however desirable [77,78]. In addition, a not negligible number of thyroid goiters are incidentally discovered during other imaging evaluations (i.e., CT, MR, PET/CT) of patients with non-thyroid indication [79]. While the performance of these imaging procedures is poor or suboptimal to identify malignant and benign nodules among adrenal thyroid incidentalomas, a significant effort should be made in the future to improve their capability to initially select patients requiring an urgent or not endocrinological evaluation combined with in-office US examination.

## 6. Conclusions

The evaluation of images has a central role in the clinical workflow. It is worth highlighting that image interpretation requires deductive reasoning, using knowledge of pathological processes, integration from prior examination and investigations and consultation with other physicians. To date, AI techniques can be an integral part of the procedure, but cannot emulate the overall process.

A further approach to improve the assessment of medical images can be represented by the integration of AI-based models with mixed reality tools. The authors retain that in-depth analysis should be performed to analyze the potential of mixed reality within the diagnostic workflow.

## Figures and Tables

**Table 1:** Machine learning (ML)-based studies.

Study	Description	Cohort	Method	Performance
Zhao et al., 2021 [21]	Classification	106 patients	SVM	Accuracy: 82%
	Benign/malignant thyroid nodules			Sensitivity: 91%
	US			Specificity: 78%
Park et al., 2019 [22]	Classification	286 patients	SVM	Accuracy: 75.9%
	Benign/malignant thyroid nodules			Sensitivity: 90.4%
	US			Specificity: 58.8%
Zhang et al., 2019 [51]	Classification	826 patients	SVM	Accuracy: 83%
	Benign/malignant thyroid nodules			Sensitivity: 86.1%
	US			Specificity: 82.7%
Yoo et al., 2018 [41]	Classification	50 patients	SVM	Accuracy: 84.6%
	Benign/malignant thyroid nodules			Sensitivity: 80%
	US			Specificity: 88.1%
Chang et al., 2016 [10]	Classification	118 patients	SVM	Accuracy: 98.3%
	Benign/malignant thyroid nodules			Sensitivity: N/A
	US			Specificity: N/A

Abbreviations: US—ultrasound; SVM—support vector machine; N/A—not available.

**Table 2:** Deep learning (DL) studies.

Study	Description	Cohort	Method	Performance
Kim et al., 2021 [59]	Malignancy risk thyroid modules	757 patients	CNN	Accuracy: 85.1%
				Sensitivity: 81.8%
				Specificity: 86.1%
Wu et al., 2021 [52]	Classification	1396 patients	CNN	Accuracy: 82%
	Benign/malignant thyroid nodules			Sensitivity: 85%
	US			Specificity: 78%
Jin et al., 2020 [11]	Classification	695 patients	CNN	Accuracy: 80.3%
	Benign/malignant thyroid nodules			Sensitivity: 80.6%
	US			Specificity: 80.1%
Liang et al., 2020 [9]	Classification	221 patients	CNN	Accuracy: 75%
	Benign/malignant thyroid nodules			Sensitivity: 84.9%
	US			Specificity: 69%
Buda et al., 2019 [57]	Nodule detection	1230 patients	CNN	Accuracy: N/A
	Predict malignancy			Sensitivity: 87%
	Risk level stratification			Specificity: 52%
Ko et al., 2019 [54]	Classification	519 patients	CNN	Accuracy: 87.3%
	Benign/malignant thyroid nodules			Sensitivity: 90%
	US			Specificity: 82%
Park et al., 2019 [22]	Classification	286 patients	CNN	Accuracy: 86%
	Benign/malignant thyroid nodules			Sensitivity:91%
	US			Specificity: 80%
Wang et al., 2019 [33]	Classification	276 patients	CNN	Accuracy: 90.3%
	Benign/malignant thyroid nodules			Sensitivity: 90.5%
	US			Specificity: 89.91%
Li et al., 2018 [55]	Classification	17 627 patients	CNN	Accuracy: 86%
	Benign/malignant thyroid nodules			Sensitivity: 84%
	US			Specificity: 87%
Chi et al., 2017 [58]	Classification	592 patients	CNN	Accuracy: 96.3%
	Benign/malignant thyroid nodules			Sensitivity: 82.8%
	US			Specificity: 99.3%
Ma et al., 2017 [56]	Classification	4782 patients	CNN	Accuracy: 83%
	Benign/malignant thyroid nodules			Sensitivity: 82.4%
	US			Specificity: 84.9%

Abbreviations: US—ultrasound; CNN—convolutional neural network; N/A—not available.

**Table 3:** Radiomics studies.

Study	Description	Cohort	Method	Performance
Park et al., 2021 [60]	Classification: Benign/malignant thyroid nodules 730 features extracted and 66 selected US	1609 patients	ML-based radiomics	Accuracy: 77.8%
				Sensitivity: 70.6%
				Specificity: 79.8%
Peng et al., 2021 [67]	Classification Benign/malignant thyroid nodules US	8339 patients	DL-based radiomics	Accuracy: 89.1%
				Sensitivity: 94.9%
				Specificity: 81.2%
Wang et al., 2021 [42]	Evaluation of extrathyroidal extension (ETE) in patients with papillary thyroid carcinoma; 479 features extracted; 10 features selected US	132 patients	ML-based radiomics	Accuracy: 83%
				Sensitivity: 65%
				Specificity: 74%
Wei et al., 2021 [61]	Evaluation of extrathyroidal extension (ETE) in patients with papillary thyroid carcinoma MRI	102 patients	ML-based radiomics	Accuracy: 79%
				Sensitivity: 75%
				Specificity: 80%
Zhao et al., 2021 [21]	Classification	106 patients	ML-based radiomics	Accuracy: 75.5%
	Benign/malignant thyroid nodules			Sensitivity: 69.7%
	US			Specificity: 78.1%
Guo et al., 2020 [64]	Prediction of thyroid cartilage invasion from Laryngeal and hypopharyngeal squamous cell carcinoma; 1029 features extracted; 30 features selected CT images	265 patients	ML-based radiomics	Accuracy: 90%
				Sensitivity: 80.2%
				Specificity: 88.3%
Kwon et al., 2020 [62]	Predict the presence or absence of BRAF proto-oncogene, serine/threonine kinase (BRAF) mutation in papillary thyroid cancer US	96 patients	ML-based radiomics	Accuracy: 64.3%
				Sensitivity: 66.8%
				Specificity: 61.8%
Wang et al., 2020 [66]	Classification	1040 patients	ML-based radiomics	Accuracy: 66.8%
	Benign/malignant thyroid nodules			Sensitivity: 51.2%
	US			Specificity: 75.8%
Zhou et al., 2020 [40]	Classification	1734 patients	DL-based radiomics	Accuracy: 97%
	Benign/malignant thyroid nodules			Sensitivity: 89.5%
	US			Specificity: 84.1%
Gu et al., 2019 [63]	Evaluating immunohistochemical characteristics in patients with suspected thyroid nodules CT images	103 patients	ML-based radiomics	Accuracy: 84%
				Sensitivity: 93%
				Specificity: 73%
Park et al., 2019 [65]	Estimate disease free survival rate in patients with papillary thyroid carcinoma;	768 patients	ML-based radiomics	Accuracy: 77%
	730 features extracted and 40 selected			Sensitivity: N/A
	US			Specificity: N/A

Abbreviations: US—ultrasound; MRI—magnetic resonance imaging; CT—computer tomography; ML—machine learning; DL—deep learning; N/A—not available.
